# Evidence-Based Review by a Multidisciplinary Team of Pediatricians on the Use of Gastric Acid-Reducing Medications in Children: Indian Perspectives

**DOI:** 10.7759/cureus.83653

**Published:** 2025-05-07

**Authors:** Uday A Pai, A.V. Ravishankar, Lalit Bharadia, Somashekara H.R., Arun Wadhwa, Baldev Prajapati, Jayakumar C., Gautam Mittal, Hrishikesh Belsare, Kanav Anand, Karthick Narayanan, Khuzema Furniturewala, Mukesh Sanklecha, Mylapore V Suresh Kumar, Piyali Bhattacharya, Prahlad N., Pramod Jog, Sanjay Wazir, Santosh T Soans, Sreenath S Manikanti, Subhashis Roy, Subhashish Bhattacharyya, Utkarsh Bansal, Vivek Goswami

**Affiliations:** 1 Department of Pediatrics, Sai Kutti Clinic, Mumbai, IND; 2 Department of Pediatrics, Kauvery Hospital, Chennai, IND; 3 Department of Pediatrics, MK Hospital, Chennai, IND; 4 Department of Pediatric Gastroenterology, Santokba Durlabhji Memorial Hospital (SDMH), Jaipur, IND; 5 Department of Pediatric Gastroenterology, Gleneagles Global Health City, Chennai, IND; 6 Department of Pediatrics and Child Health, Wadhwa Clinic, Delhi, IND; 7 Department of Pediatrics and Child Health, Gujarat Cancer Society (GCS) Medical College, Hospital, and Research Centre, Ahmedabad, IND; 8 Department of Pediatrics, Amrita Institute of Medical Sciences, Kochi, IND; 9 Department of Pediatrics, Gautam Child and Gastro Care, Patiala, IND; 10 Department of Neonatology, Belsare Children Hospital, Nagpur, IND; 11 Department of Pediatric Nephrology, Sir Ganga Ram Hospital, New Delhi, IND; 12 Department of Pediatrics, Prashant Children Hospital, Chennai, IND; 13 Department of Pediatrics, Dr. Furniturewala's Kids Care Clinic, Indore, IND; 14 Department of Pediatrics, Bombay Hospital Institute of Medical Sciences, Mumbai, IND; 15 Department of Pediatrics, Maternity, Child, and Family Health Clinic, Chennai, IND; 16 Department of Pediatric Medicine, Sanjay Gandhi Postgraduate Institute of Medical Sciences (SGPGIMS), Lucknow, IND; 17 Department of Pediatric Nephrology, Rainbow Children's Hospital, Chennai, IND; 18 Department of Family Medicine, Dr. D. Y. Patil Medical College, Hospital, and Research Centre, Pune, IND; 19 Department of Neonatology, Motherhood Hospital, Gurgaon, IND; 20 Pediatrics and Child Health, A J Institute of Medical Sciences and Research Centre, Mangalore, IND; 21 Department of Neonatology, Kauvery Hospital, Bangalore, IND; 22 Department of Pediatrics, Columbia Asia Hospital, Kolkata, IND; 23 Department of Pediatrics, CSS College, Kolkata, IND; 24 Department of Pediatrics, Hind Institute of Medical Sciences, Barabanki, IND; 25 Department of Pediatrics, Kailash Hospital, Noida, IND

**Keywords:** drug-induced dyspepsia, h2ras, on-demand use, ppis, ranitidine, stress ulcer prophylaxis, urticaria

## Abstract

Gastric acid-reducing medications (ARMs) such as proton pump inhibitors (PPIs) and histamine type 2 receptor blockers (H2 blockers) are crucial in pediatric care for treating various gastrointestinal conditions. These medications are frequently used to treat erosive esophagitis, peptic ulcer disease, and gastroesophageal reflux disease (GERD). ARMs are essential to the administration of eosinophilic esophagitis and *Helicobacter pylori* infection. Additionally, literature also supports its use in alleviating drug-induced dyspepsia, preventing stress-related mucosal damage, and lowering the risk of acid aspiration syndrome during anesthesia in critical care settings. Despite the widespread indications of ARMs, PPIs, the most potent acid suppressants, present concerns regarding safety and their inappropriate use in pediatrics. This paper aims to address these gaps by providing comprehensive, practical recommendations for ARM use in pediatric settings. The methodology involved a structured literature review and opinions from 24 pediatric specialists across India, including neonatologists, general pediatricians, pediatric gastroenterologists, a pediatric hepatologist, pediatric nephrologists, a pediatric pulmonologist, and a pediatric intensivist on the appropriate choice of ARM use in various clinical scenarios. They emphasized the benefits of H2 receptor antagonists (H2RAs) over PPIs, particularly in neonates and infants, where H2RAs offer a safer alternative due to their lower risk of adverse effects. The paper outlines the effective application of H2RAs in managing GERD, preventing stress ulcers, and treating drug-induced dyspepsia. It also provides guidelines for appropriate ARM use, stressing the need for careful patient evaluation to minimize the risk of unnecessary ARM use. Pediatricians also provided a view on the use of H2RAs beyond gastrointestinal indications, such as in urticaria, where they show promising clinical application when combined with H1-antihistamines. This paper offers valuable insights and recommendations for optimizing the use of ARM in pediatric practice. By highlighting the advantages of H2RAs and addressing the limitations and risks associated with PPIs, the paper aims to guide clinicians in making informed, evidence-based decisions. The goal is to improve clinical outcomes, promote the rational use of ARM, and enhance the quality of pediatric care.

## Introduction and background

The use of gastric acid-reducing medications (ARMs) has become essential for pediatric gastroenterology practice. Medical professionals regularly use ARMs to treat patients with gastroesophageal reflux disease (GERD) and peptic ulcer disease, as well as erosive esophagitis and eosinophilic esophagitis, and *Helicobacter pylori* infection [1]. The prevention and treatment of drug-induced dyspepsia serve as key reasons for prescribing ARMs according to medical standards [2]. The usage of ARMs reaches further than traditional gastrointestinal (GI) disorders. The prevention of stress-induced mucosal damage requires stress ulcer prophylaxis (SUP) in both Neonatal Intensive Care Units (NICUs) and Pediatric Intensive Care Units (PICUs) [3]. The preventive use of ARMs includes both anesthesia-related acid aspiration syndrome prophylaxis [4] and GERD management in asthmatic children [5]. Pediatric medical indications require thorough evaluation of treatment advantages and possible adverse outcomes.

H2 receptor antagonists (H2RAs) and proton pump inhibitors (PPIs) represent the most frequently used acid suppressant medications in pediatric medical practices today. H2RAs work by blocking H2 receptors on gastric parietal cells, thereby reducing acid secretion. The acid secretion process in parietal cells culminates with Na+, K+-ATPase enzyme activity, which is effectively blocked by PPIs, including Lansoprazole, Esomeprazole, and Pantoprazole [6]. H2RAs, including Ranitidine and Famotidine, entered the market in 1970 to provide essential management of GI conditions for adults and children. Ranitidine stands as the most commonly used H2RA medication because of its strong effectiveness combined with safety benefits and widespread pediatric application. The medical community recommends H2RAs for short-term acid suppression in pediatric GERD, as well as for managing reflux-related erosive esophagitis in infants and children, supported by a well-documented safety history [7]. The evidence shows Lansoprazole works well for children who are one year or older, but scientists have not established its effectiveness in treating neonates and infants. The past decade has brought forward mounting evidence that shows that PPI use has both reduced effectiveness and safety concerns [6]. Research has demonstrated multiple adverse effects of PPI therapy, which emerged after PPIs were declared safe [8]. Potassium‐competitive acid blockers (PCABs) stand as a new ARM class that gained approval for adult erosive esophagitis treatment but continues to be studied for different therapeutic applications [9]. PCABs remain unapproved for pediatric use because research about their effectiveness in children is insufficient. H2RAs function as a well-established and safer alternative acid reflux medication for pediatric patients of all age groups because PPI therapy has safety concerns and approval restrictions for pediatric practice, and PCABs lack current pediatric approval. H2RAs provide pediatric care with a flexible therapeutic choice because they offer numerous clinical applications that extend past GI conditions.

Unmet needs and scope 

The extensive use of ARM continues to generate discussion about its safety and effectiveness, and proper usage in younger patients [10]. Research shows a growing issue of wrong ARM medication prescriptions in pediatric healthcare facilities where doctors prescribe these drugs outside their approved uses [11]. Pediatric patients who receive excessive or improper administration of ARMs face substantial adverse effects, which include minor GI problems, together with serious conditions such as GI infections, bone fractures, and kidney injuries [12]. The diagnostic similarities between GER and other conditions create a high risk for unnecessary ARM prescriptions that affect infants and neonates particularly badly [13].

Primary care physicians, along with young pediatricians, face difficulties deciding about ARM prescriptions for children because practice-based guidelines remain unclear. The Indian pediatric healthcare system faces multiple challenges because of its varied patient population and infrastructure conditions, and clinical practice patterns, which both create difficulties and potential benefits regarding ARM prescription and use.

The evidence-based review paper EMPACIP (Evidence-Based Review by a Multidisciplinary Team of Pediatricians on the Use of Gastric ARMs in Children: Indian Perspectives) relies on extensive research in various pediatric specialties. The paper enhances pediatric ARM therapy selection and use through appropriate indication identification and inappropriate use management, and risk assessment. The research intends to bridge existing knowledge gaps regarding ARM applications in pediatric medical practice. The evidence-based guidance document includes specific recommendations that will assist primary care practitioners in making their clinical choices based on sound data while reducing risks and enhancing pediatric care quality.

Objectives

This paper presents a comprehensive, practical approach for the use of ARM in standard pediatric care across India. It draws on recommendations from distinguished Indian pediatric specialists to provide evidence-based guidance on ARM use in children. This paper presents recommendations for the use of ARMs in the prevention and management of drug-induced dyspepsia in children.

A total of 24 pediatric specialists from India collaborated for this evidence-based review paper through their representation as neonatologists, general pediatricians, pediatric gastroenterologists, a pediatric hepatologist and gastroenterologist, pediatric nephrologists, a pediatric pulmonologist, and a pediatric intensivist. The team initiated the project by holding a virtual session to define the research boundaries and create a systematic approach for literature investigation. The team performed an extensive structural review of existing literature to guarantee that all recommendations were based on current evidence. A physical meeting of pediatricians was held on May 12, 2024, in Mumbai to review current practices and evidence-based data regarding drug-induced dyspepsia and the use of ARMs. The team assessed the severity of drug-induced dyspepsia in pediatric practice using the *5-point Likert Dyspepsia Severity Scale* through polling methods [14]. The online platform *Mentimeter* was used for conducting the polling activities.

## Review

Reviewing the use of PPIs in pediatric practices

PPIs are among the most widely prescribed medications globally, both in adults and children. Although there is ample evidence establishing its place in therapy for use in adults, its use in pediatrics is based on little evidence or evidence extrapolated from trials in adults. Despite their limited indications, PPI use has significantly increased among pediatric populations. A significant proportion of prescriptions in children are generated for off-label indications [15]. Considering the lack of strong evidence and rising concern about the side effects of PPIs, it is important to strictly limit their use to approved indications where clear benefits are indicated. PPIs are currently approved only for use in children over the age of one year [12,15]. Specifically for the short-term treatment of symptomatic GERD, peptic ulcer disease, healing of erosive esophagitis, eosinophilic esophagitis (EoE), and the eradication of *H. pylori* [12,15]. The indications for PPI use in infants under one year old are less clear and are considered off-label. CONFOR, a recently published consensus document by a multidisciplinary team on rationalizing the use of ARMs, has listed multiple inappropriate indications of PPI in children and adults [12]. These indications include routine use in patients with mild and infrequent heartburn, nonspecific upper abdominal discomfort without a confirmed diagnosis, routine prophylactic use in primary care for non-ulcerogenic conditions, prophylactic use alongside Non-Steroidal Anti-Inflammatory Drugs (NSAIDs) and antibiotics, stress ulcer prophylaxis (SUP) in low-risk patients and those hospitalized outside the intensive care unit (ICU), and long-term use without reevaluation, among others [12]. Current guidelines suggest that a PPI trial in infants should be considered only after a referral to a pediatric gastroenterologist and the failure of first-line non-pharmacological treatments, such as thickening of feeds and avoiding overfeeding, and second-line strategies, such as a trial of cow's milk elimination, allergy immunology consultation, etc. [12,15]. Despite the limited number of approved indications for the use of PPIs in children and clear recommendations from different guidelines, inappropriate prescription of PPIs is rampant across this age group. A study evaluating the prevalence and appropriateness of PPI use in children found that 86.2% of PPI prescriptions in children were inappropriate, with co-prescription with steroids or NSAIDs being a major cause reported in 28.7% of the cases [11]. In the study population, 58.4% of children prescribed PPIs had no comedication or diagnosis necessitating PPI use. The study outcomes highlighted that PPIs are prescribed in a blanket manner even in pediatric practices, and there is a need for efforts directed towards promoting their rational use [11].

The use of ARM may be effective only in infants with erosive esophagitis. However, their use in infants with symptoms attributed to GERD has significantly increased despite limited approval and evidence for their safety and efficacy in this age group [16]. Although PPIs are not approved for children under one year, they are frequently used in clinical settings despite guidelines advising against their routine use due to insufficient clinical evidence and demonstrated lack of efficacy in young infants [17]. The dosing and duration of PPIs in infants are unclear and are not well studied; still, PPIs are often administered at higher doses than necessary, with no significant clinical benefit demonstrated in this patient population [16,18]. There is a concerning trend towards overtreatment with ARM therapy, specifically PPIs, in both term and preterm infants based on symptom presentation alone, without objective diagnostic measures such as pH monitoring or endoscopy, and despite a lack of data on pharmacological management and potential risks [16]. Recent findings suggest that many infants with nonacid GER can cause more distress and crying than acid GER, and thus, PPIs may not alleviate symptoms as expected [16,19]. Also, for clinical action, PPIs should be administered 30-60 minutes before feeding, which is impractical for young infants due to their feeding and sleeping patterns [19]. Additionally, the liver enzymes responsible for metabolizing PPIs mature only around 5-6 months of age. Infants also have different pH and enzyme profiles compared to adults, which affects drug metabolism and efficacy [19].

The rational and appropriate use of PPIs has also become highly important as our understanding of their associated side effects continues to grow. The long-term safety of PPIs is a matter of concern, especially in pediatric populations [20]. Chronic PPI usage has been linked with a wide range of serious side effects, including increased risk of GI and lower respiratory tract infections, bone fractures, and allergies [12,15]. Long-term PPI use results in dysbiosis, altering the microbiome in the oral cavity, gut, and lungs, which is further associated with necrotizing enterocolitis, late-onset sepsis in premature infants, Clostridium difficile infection (CDI), asthma, obesity, and small intestinal bacterial overgrowth (SIBO) [21]. In a population-based study in infants and children, PPI use was found to be associated with a higher risk of CDI compared to H2RAs [22]. Studies also showed that PPI use in children increases the risk of serious infections affecting multiple systems, including the digestive, respiratory, urinary, and nervous systems, and heightens the risk of both bacterial and viral infections [23]. Long-term PPI use increases the risk of childhood fractures by reducing calcium absorption and bone remodeling, with studies showing a 20% higher fracture risk compared to H2RAs [8,24]. PPI-induced hypochlorhydria raises the risk of allergies, including food and medication allergies, anaphylaxis, and asthma [25]. PPIs may increase asthma risk by disrupting gut and lung microbiomes, triggering immune responses, and airway inflammation [26]. PPIs are linked to a 57% higher asthma risk in children due to disruptions in gut and lung microbiomes [27]. Additionally, PPI use in hospitalized children is reported to have a 37% higher risk of acute kidney injury, likely due to mechanisms such as interstitial nephritis and oxidative stress [28,29]. Overall, the evidence strongly supports the need for appropriate use of PPIs in pediatric settings, avoiding their use without clear indications. Given the uncertainties about the safety of PPIs in children, their use should be restricted to those above one year of age [12].

While PPIs have a role in managing certain pediatric conditions, their use should be judicious and restricted to clear, evidence-based indications. Due to potential adverse effects and limited safety data, especially in infants under one year, their use should be restricted to cases where non-pharmacological treatments fail and the benefits outweigh the risks. The use of PPIs in NICU settings was particularly cautioned against due to the increased risk of side effects. Healthcare providers must follow guidelines to avoid the unnecessary and potentially harmful use of PPIs in young patients.

Specialist recommended individualized assessment, prioritization of non-pharmacologic interventions, and cautious ARM administration to ensure clear benefits while minimizing risks. H2RAs like ranitidine may be a safer alternative to PPIs in this population.

Reviewing the use of H2RAs in pediatric practices

GI Uses of H2RAs in Neonates and Infants

Gastroesophageal reflux (GER) is a common, self-limiting condition in infants due to developmental factors such as an immature lower esophageal sphincter and delayed gastric emptying [30,31]. Common GER symptoms in infants include frequent regurgitation, vomiting, coughing, choking, hiccups, irritability, and crying [16,32]. Although regurgitation is common in neonates, typically occurring several times a day, it resolves naturally by 6-12 months as the infant matures [32,33]. Distinguishing GER from GERD is challenging due to nonspecific symptoms and unreliable diagnostic criteria [33]. Overdiagnosis of GERD, particularly in NICU infants, can lead to unnecessary acid suppression therapy [32,33]. Current guidelines recommend avoiding diagnostic tests and treatment in the absence of red flags or significant impact on growth and development [34]. While high-risk infants with persistent symptoms require thorough evaluation [33].

Empiric pharmacological treatments, including ARMs and prokinetics, are not recommended in neonates and infants in the NICU [13]. Guidelines suggest that pharmacological management with ARM should be reserved for severely symptomatic infants after diagnostic workups who have not shown improvement with nonpharmacologic management [18]. Also, ARM should only be continued with clear benefits and close monitoring, and attempts should also be made to discontinue the treatment when unnecessary. Continuing ARM therapy after discharge from the NICU in the absence of benefit may lead to unnecessarily prolonged treatment and an increased risk of adverse effects [35]. A report documented that nearly 50% of preterm babies born at <28 weeks' gestational age are prescribed ARM, while a quarter of them are continued on ARM therapy after discharge [36]. Studies have found no substantial link between GERD and cardiorespiratory events in preterm infants, yet many neonatologists prescribe ARM to manage apneas despite recommendations to reserve these treatments for cases with documented pathological acid reflux [16].

Now, it is a well-known fact that premature infants have nonacid or weakly acidic reflux rather than acid reflux [37]. For infants with weakly acidic or non-acidic refluxate, acid suppression is ineffective as the pH of reflux is already >6.0 [32]. Current protocols for pharmacological treatment of GERD are primarily based on studies in adult and older children, where symptoms are more directly linked to acid reflux. The use of ARM in neonates also increases the risk of pneumonia, respiratory events, and mortality [38]. Studies have shown that ARAs increase the risk of GI infections in term infants and necrotizing enterocolitis in preterm infants. ARM use is also linked to ventilator-associated pneumonia in the PICU and late-onset sepsis in the NICU. Additionally, in healthy infants in outpatient settings, there are reports that the incidence of community-acquired pneumonia increases with ARM use [39]. Given the growing evidence for potential adverse effects, ARMs should only be prescribed to neonates and infants after careful consideration of the benefit and risk balance.

SUP is a critical component of care in NICUs and PICUs, especially for critically ill children. It has been found that stress-related mucosal damage and upper GI bleeding may complicate critical illness in up to 25% of infants and children [7]. The incidence of upper GI bleeding is significantly higher in children requiring mechanical ventilation [40]. Key risk factors include coagulopathy, organ failure, and high Pediatric Risk of Mortality (PRISM) scores (≥10) [40]. An intragastric pH > 4 is protective against stress ulcer formation. ARMs can prevent stress ulcers by maintaining this protective gastric pH [40]. Clinically, SUP is defined as the use of a PPI, an H2RA, or sucralfate within the first two days of PICU admission among children who had not been on these drugs at home and had no evidence of GI bleeding [3]. According to reported literature, the most common indications for SUP include patients receiving invasive mechanical ventilation, those expected to be ventilated for two or more days, and those on mechanical ventilation but nil per os (NPO). SUP is typically discontinued when patients are no longer on mechanical ventilation, are no longer on NPO, or are discharged from the intensive care unit. However, these indications are primarily derived from adult studies, with limited data on definitive SUP indications in critically ill children [41]. The use of SUP is not without risks. It increases the risk of ventilator-associated pneumonia (VAP) and C. difficile-associated diarrhea due to altered gastric acidity [40]. A retrospective study of 30,177 PICU patients suggests that routine SUP may be unnecessary in children with corticosteroid exposure. The authors of this study advocated selective use of SUP based on individual GI bleeding risk factors [42]. Another descriptive study also highlighted the importance of assessing known risk factors for stress ulcer-related GI bleeding before considering the blanket administration of SUP in children hospitalized for acute severe asthma [43].

PPIs are the most frequently used ARM for SUP, even in critically ill children, although it is not the approved indication [40]. Also, there is moderate-quality evidence that supports PPI use for SUP. PPIs have been associated with an increased risk of central line-associated bloodstream infections (CLABSI) and pneumonia in critically ill children. Considering this, when the benefits are weighed against the potential risks, the routine use of PPIs for SUP in critically ill children raises concerns [40,44]. On the other hand, studies have demonstrated the effectiveness of H2RAs like ranitidine in the prevention of stress-induced gastric mucosal lesions and gastric bleeding in critically ill pediatric patients. In a prospective, randomized study of 53 mechanically ventilated newborns, short-term prophylactic ranitidine significantly reduced stress-induced gastric mucosal lesions. Endoscopic evaluation showed fewer lesions in the ranitidine group compared to controls, with no ulcers observed in the treated infants. Logistic regression confirmed a decreased risk of lesions with ranitidine (odds ratio [OR] 0.03, 95% confidence interval [CI] 0.003-0.178). These results suggest that prophylactic ranitidine effectively prevents stress-induced gastric lesions in newborns [45]. A study evaluating ranitidine for stress-associated gastric bleeding in critically ill neonates found faster bleeding control in the ranitidine group compared to controls, with no adverse effects reported [46]. Similarly, another randomized, controlled trial showed that ranitidine reduced the rate of upper GI bleeding in critically ill children [47]. Studies also indicated that ranitidine significantly reduces the incidence of upper GI bleeding without increasing risks for pneumonia [48]. Based on these results, ranitidine can be recommended for managing stress-related gastric bleeding in neonates [46].

ARMs are commonly prescribed in NICUs for conditions such as stress-related mucosal disease, coagulopathy with gastric bleeding, prolonged ventilation (>48 hours), and neonates with spinal cord injury, multiple trauma, hepatic failure, or severe thermal injury. However, they emphasize the limited clinical data supporting ARM use in neonates. While ARMs play a role in preventing stress ulcers in critically ill children, their use should be carefully assessed based on individual risk factors such as illness severity, coagulopathy, mechanical ventilation duration, history of GI bleeding, and concurrent medications. This approach minimizes unnecessary exposure to adverse effects while ensuring effective prophylaxis for high-risk cases. Beyond SUP, it is recommended ARMs only for upper GI bleeds unrelated to Vitamin K deficiency and for symptomatic reflux with excessive irritability, arching, disease progression, or failure to thrive. Apnea alone is not a valid indication. In suspected GERD with marked symptoms and no impedance monitoring available, a one-week ARM trial may be considered; continued use should depend on clinical response and pediatric gastroenterology consultation. Routine ARM use in ventilated neonates is strongly discouraged. It is recommended that individualized assessment, prioritization of non-pharmacologic interventions, and cautious ARM administration be used to ensure clear benefits while minimizing risks. H2RAs like ranitidine may be a safer alternative to PPIs in this population.

GI uses of H2RAs in toddlers, older children, and adolescents

On-Demand Use for GI Conditions

As-needed or on-demand treatments that provide prompt symptom relief are important in clinical practice [49]. Ideally, effective on-demand therapies should have a rapid onset of action to provide faster symptom relief [7,50]. H2RAs, like ranitidine, are favored as on-demand therapy due to their rapid onset of action, which quickly increases gastric pH and alleviates the symptoms. These drugs also have high bioavailability from the first dose, further enhancing their effectiveness for immediate relief. Ranitidine acts uniformly on parietal cells, inhibiting gastric acid secretion promptly within 4 hours of administration, even when taken after meals [49]. In contrast, PPIs require administration 30 to 60 minutes before meals for optimal efficacy [7]. PPIs bind only to the proton pumps in immature and active parietal cells, requiring longer to fully inhibit acid secretion, and need approximately three days to reach a steady state. Hence, PPIs have a slower onset of action and do not provide immediate relief [49,51]. Clinical studies have demonstrated the rapid onset of action of ranitidine in children. In a randomized, double-blind study, a single dose of 75 mg oral ranitidine administered to children aged 4-11 years with symptoms of GERD led to a rise in intragastric pH approximately 30 minutes after dosing [52]. Based on current literature in adults and children, a consensus paper recommended ranitidine for immediate relief in pediatric patients with gastritis, emphasizing its suitability for on-demand therapy compared to PPIs [7].

Nocturnal Acid Breakthrough

Nocturnal acid breakthrough (NAB) is characterized by the presence of intragastric pH < 4 for at least 60 continuous minutes during the overnight period (10:00 PM to 6:00 AM) [53]. There are reports of the high prevalence of NAB among children despite treatment with twice-daily PPIs. A study conducted among children 1 to 13 years of age with esophagitis showed that nearly 90% of the children experienced nocturnal acid reflux despite being on twice-daily PPI therapy [54]. Administration of ranitidine at bedtime has proven more effective than bedtime PPI in preventing nocturnal acid reflux in adult studies. However, there is a paucity of literature reporting the efficacy of nocturnal ranitidine/H2RA in pediatric settings [7]. In a consensus document from India, pediatricians recommended bedtime ranitidine for pediatric patients managing nocturnal acid reflux based on their clinical experience [7]. Additionally, the API-ISG consensus guidelines for managing GERD recommend adding an H2RA at bedtime for patients experiencing nocturnal reflux symptoms despite PPI use [20].

On-Demand Use as Part of the ARM Deprescribing Algorithm

With increasing awareness about the risk of ARM, especially PPI-associated side effects, steps are being taken to deprescribe inappropriate long-term PPI use. Patient-centric PPI deprescribing guidelines suggest that patients without a clear indication of chronic PPI use should be considered for deprescribing. Apart from PPI cessation, PPI dose tapering, and replacement of PPIs with H2RAs, one of the strategies to deprescribe PPI therapy is on-demand or as-needed acid suppression therapy, either PPI or H2RA [12]. According to a study evaluating deprescribing non-indicated long-term PPI use, prescribing H2RAs instead of PPIs as a first step is a patient-centered approach through on-demand regimes [55]. On-demand H2RA use is a successful PPI de-escalation strategy, and on-demand ranitidine can be considered as a step-down therapy when symptoms present after tapering off the PPI [56]. By extrapolating the clinical evidence in adult patients, on-demand H2RAs can be considered for deprescribing PPIs in children.

Ranitidine provides rapid and effective symptom relief, making it a preferable choice for on-demand ARM in children. H2RAs are a better option compared to sudden, occasional heartburn or acid reflux. Its quick action is useful for managing NABs when on PPI therapy. Ranitidine is also effective as a step-down therapy for PPIs. When long-term PPI use needs to be reduced, ranitidine helps maintain symptom control and prevents rebound acid hypersecretion. Additionally, ranitidine is valuable for immediate symptom relief when PPI doses are missed. It offers a practical solution to quickly alleviate symptoms if a PPI dose is skipped.

*Prophylaxis of Acid Aspiration Syndrome* 

Acid aspiration syndrome (AAS) represents a serious complication associated with general anesthesia, originating from the aspiration of acidic gastric contents into the lungs. This typically occurs when laryngeal reflexes are suppressed during anesthesia. The risk of AAS is heightened by the acidity and volume of gastric contents, with the syndrome being characterized by the aspiration of more than 25 ml of gastric fluid with a pH below 2.5 [4,57]. Regurgitation or aspiration can occur at any stage of anesthesia, including induction, maintenance, or reversal, and is exacerbated by inadequate anesthesia depth or existing airway disorders [7,57].

Preventative measures against AAS include fasting, gastric decompression, accelerated gastric emptying, and airway protection. Additionally, one of the strategies is to modify the pH and volume of gastric secretions before anesthesia. This can be achieved using ARMs like H2RAs or PPIs, which reduce both the volume and acidity of gastric fluid, thereby mitigating the risk of acid aspiration [4,57]. However, there are differences in the effects of H2RAs and PPIs on gastric secretions, with evidence favoring H2RAs for AAS prophylaxis [57]. It is well known that H2RAs like ranitidine effectively inhibit gastric acid secretion and reduce gastric volume. Earlier studies have shown that oral administration of ranitidine before anesthesia induction significantly increases gastric pH and decreases gastric volume in children [7]. Evidence suggests that when administered in two doses (the night before and on the morning of surgery) or when the drugs were given intravenously, the effect of PPI and H2RA is similar. However, a single oral dose of H2RA immediately before surgery is significantly more effective than a PPI [4]. An older study involving fifty healthy children (aged 8 months to 11 years) found that ranitidine at the dose of 2 mg/kg significantly increased the incidence of gastric pH above 2.5 compared to the control group. Ranitidine treatment also resulted in a mean gastric aspirate volume of 2.76 ± 0.76 mL, significantly lower than that of the control group (*P *< 0.001) [58]. A randomized trial conducted among 75 children undergoing elective surgery showed that 5 ml/kg of plain water and 2 mg/kg of ranitidine administered orally for three hours before surgery modifies gastric fluid volume and pH, minimizing the risk of aspiration pneumonitis [59]. Another study evaluating the effect of oral liquids and ranitidine in children undergoing outpatient surgery also showed that ranitidine 2 mg/kg with or without fluids decreases both the volume and pH of gastric contents [60]. According to a meta-analysis of randomized controlled trials, ranitidine is more effective than PPIs as premedication before surgery in reducing the volume of gastric secretions (by an average of 0.22 mL/kg) and increasing gastric pH (by an average of 0.85 pH units) [57].

Considering the current clinical evidence, it is recommended that ranitidine be used as the prophylactic agent of choice to reduce the risk of AAS in children undergoing anesthesia. Compared to PPIs, ranitidine demonstrates superior efficacy in increasing gastric pH and decreasing gastric fluid volume when administered as a single oral dose shortly before surgery. Its proven efficacy in altering gastric fluid characteristics aligns with best practices for minimizing the risk of acid aspiration and ensuring safer surgical outcomes. Therefore, incorporating ranitidine into preoperative care protocols offers a reliable strategy for minimizing the risk of AAS and enhancing patient safety during anesthesia.

Management of GERD in Children With Asthma

GERD-related extra-esophageal (EE) manifestations are common and present significant diagnostic and therapeutic challenges. Among these, asthma is one of the most frequent extra-esophageal manifestations of GERD, particularly in children [61]. On the other hand, epidemiological data suggest that nearly 80% of asthma patients also experience GERD symptoms, including heartburn and regurgitation [62]. The reported prevalence of GERD in children with asthma varies widely from 19.6% to 80.0%, largely due to differences in diagnostic criteria for GERD (symptoms vs. esophageal pH monitoring), asthma severity, and study design (prospective vs. retrospective) [63]. GERD and asthma can trigger each other, though the exact relationship between the two remains unclear. GERD-induced bronchoconstriction is explained by two theories: *Reflux Theory* and *Reflex Theory*. According to *Reflux Theory*, reflux directly affects the lungs, while according to *Reflex Theory*, esophageal reflux triggers bronchoconstriction via vagal nerves [5,64]. On the other hand, asthma can provoke reflux through several mechanisms, including an increased gastroesophageal pressure gradient from increased negative intrathoracic pressure during inspiration due to airflow obstruction [62-64]. In addition, the medications used to treat bronchial asthma, including β-adrenergic agonists, theophylline, and high doses of oral corticosteroids, can aggravate reflux [62]. Recent studies have also established a relevant role for esophageal motility and neuronal sensory abnormalities in linking these two diseases [65]. Initial diagnosis in asthmatic children with suspected GERD involves empiric PPI therapy. If there's no response and no regurgitation, GERD is unlikely, and PPIs are stopped. Acid-suppressive therapy is used for confirmed GERD [64]. The 24-hour pH impedance monitoring, while sensitive, is limited in children and should be used for extra-esophageal GERD cases unresponsive to PPIs [63].

Mainly, two acid-suppression therapies have been described in the literature and guidelines as an effective therapy for GERD-related symptoms in asthmatic patients, which include H2RAs and PPIs [5]. Although PPIs are routinely prescribed for asthmatic children with GERD, studies indicate that PPI treatment in children with asthma is often unsuccessful and commonly associated with adverse effects, such as an increased risk of symptomatic respiratory infections. Nearly half of the children with asthma and GERD are asymptomatic, and PPIs at approved doses do not improve asthma outcomes in children with poorly controlled asymptomatic GERD by inhaled corticosteroids [64]. Also, there is no strong literature to support the treatment of GERD to improve asthma control, and the role of GERD therapy in improving asthma-related objective measures is controversial [62]. Many studies have failed to show improvements in asthma symptoms with the use of PPIs [5]. A trial conducted by the American Lung Association Asthma Clinical Research Centers also did not show any benefit of PPI therapy concerning the rate of asthma attacks, asthma symptoms, nocturnal awakening, quality of life, or lung function in asthma patients without concomitant reflux symptoms. Similar outcomes were observed in other studies [62]. According to the GINA Report 2024, patients with poorly controlled asthma should not be treated with ARM unless they also have symptomatic reflux [66].

Empiric ARM therapy, particularly PPIs, is often used for diagnosing GERD, but its impact on asthma control is inconsistent, with many studies showing no significant improvement in asthma outcomes. Given the potential adverse effects of PPIs, including increased risk of respiratory infections and the lack of robust evidence supporting routine PPI use in asymptomatic GERD, H2RAs may be a safer alternative in the management of GERD in asthmatic children. ARM therapy should be reserved for asthmatic children with symptomatic GERD rather than used routinely, aligning with recent guidelines that emphasize targeted treatment based on symptom presentation.

*Management of Drug-Induced Dyspepsia* 

Drug-induced GI symptoms, including upper abdominal pain, early sense of satiety, epigastric discomfort or pain in the upper abdomen or behind the breastbone, flatulence, nausea, and vomiting, are among the most commonly observed side effects often reported by patients after initiation of treatment [67]. These drug-induced GI symptoms are dose-dependent and individual-specific. Age, sex, genetic, and physiological factors are among the factors that define the individual's susceptibility to drug-induced side effects. Pre-existing conditions or diseases may also predispose individuals to these side effects [68].

Drugs are reported to be a frequent cause of new-onset dyspeptic symptoms. Although it is not clearly understood, direct mucosal injury, alterations in gastric motility, induction of gastric acid rebound, and provocation of GER are the key proposed mechanisms of drug-induced dyspepsia [68]. Assessing the prevalence of dyspepsia as an adverse effect of a drug is not easy, as there is a high frequency of the symptoms defining dyspepsia in the general population, and also, many patients are prescribed more than one drug [68]. It is also difficult to distinguish between dyspepsia as a drug-induced side effect and spontaneously occurring symptoms [67]. Also, there is significant variation in the reported prevalence of drug-specific dyspepsia due to the lack of a uniform definition for diagnosing drug-induced dyspepsia and the use of varying criteria in studies [69].

According to ROME IV criteria for the diagnosis of functional dyspepsia in adults, one or more of the listed symptoms must be present for at least the past three months, with symptoms starting at least six months before diagnosis. These symptoms include bothersome postprandial fullness (three days per week), early satiety (three days per week), epigastric pain (one day per week), and epigastric burning (one day per week) with no evidence of structural disease [70]. For children and adolescents, the ROME IV criteria for functional dyspepsia are slightly different. The criteria require one or more bothersome symptoms from postprandial fullness, early satiation, or epigastric pain or burning not associated with defecation to be present for at least two months before diagnosis. Additionally, any other medical conditions should be ruled out through appropriate evaluation [71]. Evidence suggests that the majority of children with dyspepsia do not exhibit mucosal lesions on endoscopy; therefore, endoscopy is not mandatory for diagnosing functional dyspepsia in children [2]. ROME IV criteria do not define functional dyspepsia in infants, as it is not commonly reported in this set of population [72]. While the ROME IV criteria provide a framework for defining functional dyspepsia, they do not specify criteria for drug-induced dyspepsia; hence, defining drug-induced dyspepsia remains challenging. Currently, there is no definition or criteria for the diagnosis of drug-induced dyspepsia in adults, as well as in children and adolescents, highlighting the need for developing a definition of drug-induced dyspepsia in both adults and children.

**Figure 1:**
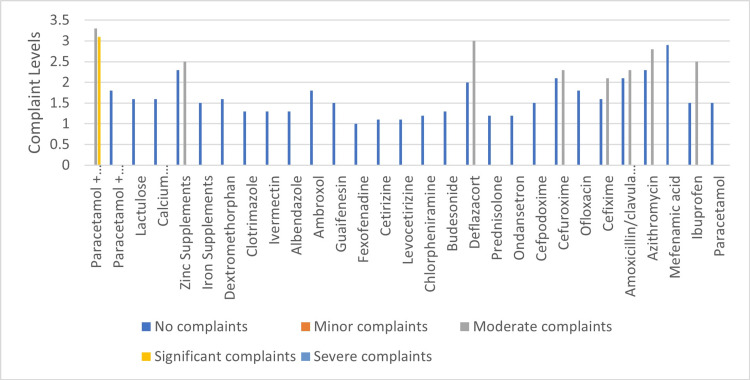
Drug-induced dyspepsia severity scoring for most commonly prescribed drugs and combinations in pediatric practice. Image credit: All authors. Note: Placetamol+ appears twice on the vertical axis. The top label refers to the standard formulation, while the bottom refers to a variant (extended-release).

The pediatricians during the discussion noted that upper abdominal discomfort or pain and nausea are the most common symptoms of drug-induced GI discomfort in pediatric patients. Based on available data and pediatricians' clinical experience, the most commonly prescribed drugs and their combinations in children were identified. Using a 5-point Likert scale (score 1: no complaints, score 2: minor complaints, score 3: moderate complaints, score 4: significant complaints, score 5: severe complaints) [14], pediatricians evaluated the severity of dyspepsia for the commonly prescribed drugs. The average severity scores based on polling are presented in Figure 1.

NSAIDs are among the most widely prescribed medications in both adults and children, which are frequently associated with dyspeptic symptoms, including epigastric pain, nausea, and a feeling of heaviness in the epigastrium [73,74]. The risk of dyspepsia with NSAIDs varies by specific drug and dosage, and the risk increases with prolonged use or higher doses [75]. NSAID-induced dyspepsia is not fully explained by peptic ulcers alone and may also involve erosive esophagitis, altered gut permeability, and changes in gastric mechanosensory function [69]. Other medications frequently causing dyspepsia include corticosteroids, antimicrobials, and high doses of iron and zinc supplements. Corticosteroids and antimicrobials, as well as higher doses of iron and zinc, are known to cause dyspeptic symptoms, though the literature on this is limited [68]. As seen in Figure 1, ibuprofen, mefenamic acid, azithromycin, amoxicillin/clavulanic acid, cefuroxime, deflazacort, iron, and zinc supplements were scored above two on the scale of drug-induced dyspepsia severity score. Also, prednisolone and a combination of paracetamol with ibuprofen and mefenamic acid scored above three on this scale.

Drug avoidance, if possible, would be an ideal step in the management of drug-induced dyspepsia. While ARMs and prokinetics are commonly used as first-line therapies for managing dyspepsia in children. ARMs are preferred when epigastric pain is the predominant symptom, while prokinetics may be chosen when postprandial fullness or early satiety is the main symptom [2]. It is suggested that, in patients with a previous history of peptic ulcer disease, ARMs can be co-prescribed with the drugs having a high risk of dyspepsia [69]. Although PPIs are largely prescribed as prophylaxis as well as treatment for drug-associated GI symptoms, including dyspepsia, they lack solid evidence to support their routine use for this indication in children. Guidelines recommend that PPI prophylaxis should only be considered in high-risk individuals, defined as those with a history of ulcer bleeding, old age, or comorbid illness, which are conditions rarely seen in children [11]. Also, co-prescription of PPIs, mainly with NSAIDs and steroids, is reported to increase the risk of side effects and complications. PPIs can alter the composition of the gut microbiome, which is reported to worsen NSAID-induced small intestine injury. The PPI-associated dysbiosis also increases the incidence and severity of NSAID enteropathy, further questioning the safety of PPI co-prescribed with NSAIDs in children [76]. Corticosteroids are known to cause hypocalcemia and increase the risk of fractures. They also affect the function and survival of osteoblasts and osteocytes. Additionally, the use of corticosteroids is associated with an elevated risk of infections [77]. As mentioned earlier, PPIs also affect bone metabolism by decreasing calcium absorption and increasing osteoclastic activity, which further adds to the fracture risk associated with corticosteroids. Also, PPI-induced dysbiosis further adds to the increased risk of infections associated with corticosteroids. Hence, in pediatric settings, PPIs should be cautiously co-prescribed with NSAIDs and steroids for management of GI side effects. Here, H2RAs are promising alternatives to PPIs for the management of dyspepsia, especially in children.

Pediatricians proposed defining drug-induced dyspepsia in children based on Rome IV criteria for functional dyspepsia. It is characterized by bothersome postprandial fullness, early satiety, epigastric pain, or burning following the initiation of a new medication. However, pinpointing symptom onset remains challenging due to drug variability. Routine prophylactic use of ARMs with dyspepsia-inducing medications is not recommended. Instead, ARMs should be used as needed, starting when symptoms arise and stopping within 72 hours of resolution. H2RAs are preferred over PPIs due to their rapid action and safety profile. Non-pharmacological strategies, such as dietary adjustments, should complement pharmacologic management. Further research is needed to assess prescribing practices, prevalence, and the appropriateness of routine ARM therapy for pediatric drug-induced dyspepsia.

Non-GI Uses of H2RAs

H2RAs, though traditionally used as gastric acid-reducing medication, also have therapeutic applications beyond GI conditions in children. These non-GI indications primarily involve the treatment of allergic conditions, such as urticaria.

Managing Chronic Urticaria

Urticaria, or hives, is a prevalent skin condition characterized by pruritic, transient wheals that typically resolve within 24 hours [78,79]. Urticaria is classified as acute or chronic, with chronic cases lasting more than six weeks and subdivided into chronic spontaneous or chronic inducible forms [79]. Childhood urticaria has a reported prevalence of 2%-6%, with acute cases being more common. Chronic urticaria, though less frequent, has a greater impact due to recurrence and often unknown causes [79,80]. Common triggers for urticaria in children include infections, medications, and foods, with infections being a more frequent cause of urticaria in infants and children compared to adults [79,81].

H1-antihistamines are the standard treatment for symptomatic treatment of both acute and chronic urticaria in adults as well as children. H1-antihistamines help relieve itching and reduce the number, size, and duration of urticarial lesions. However, since histamine effects are mediated through both H1 and H2 receptors, and other vasoactive substances may also be involved, relief may not be sufficient with H1-antihistamines alone [82]. Some patients may experience more severe allergic reactions or recurrent manifestations even after initial treatment with an H1 antihistamine [83]. Approximately 50% of patients do not respond to the initial dose of H1-antihistamines, and a subset may become refractory to treatment even at higher doses [84].

Given the involvement of both H1 and H2 receptors in many pathological processes, the combination of H1 and H2 antihistamines has been explored for treating histamine-mediated disorders, including urticaria [83]. Evidence suggests that adding an H2-antagonist to an H1-antihistamine may provide moderate additional relief from itching and wheal formation in patients resistant to H1-antihistamines alone [82,83]. Multiple studies have demonstrated that this combination can improve clinical outcomes in patients with acute allergic disorders and more severe cases [83]. There is enough evidence to support the use of this combination in acute cases of allergic disorders, including acute urticaria; however, there is no extensive literature on its use in chronic recurrent cases of urticaria. Historically, the combination of H1 and H2 receptor antagonists has been used since 1978 for chronic urticaria unresponsive to conventional therapy, showing effectiveness in some cases [85]. Early studies from the 1980s indicated the benefits of combining chlorpheniramine with cimetidine or ranitidine with diphenhydramine [86-88]. A retrospective study from Japan also showed moderate symptom improvement with lafutidine in chronic urticaria resistant to H1-antihistamines [89]. However, a Cochrane review of four studies on H1-H2 antihistamine therapy concluded that evidence supporting H2-antagonists as an add-on therapy is insufficient, particularly due to the small size and low quality of the studies [90].

Current guidelines from EAACI/GA2LEN/EDF/WAO recommend second-generation non-sedating H1-antihistamines as first-line treatment for urticaria. For chronic urticaria unresponsive to standard doses, the guidelines suggest increasing the dose of the H1-antihistamine before considering alternative treatments such as omalizumab [91]. The addition of treatment other than H1-antihistamine is suggested if inadequate control on a high dose after 2-4 weeks or earlier [91]. Although the routine use of H2RA is not strongly recommended by EAACI/GA2LEN/EDF/WAO guidelines, the guidelines suggest that H2RAs may be considered for individual patients in specific clinical situations and due to their cost-effectiveness compared to omalizumab [91]. The 2022 Skin Allergy Research Society's Guideline from India also suggests H2RAs as an alternative to omalizumab for patients not responding to high doses of H1-antihistamines [92]. In some guidelines, it is recommended to discontinue the H2-antagonist if there is no improvement in clinical symptoms within two to four weeks after initiation [93]. The current recommendations for a combination of H1- and H2 antihistamines are mainly based on studies conducted in adults, as there is little or no data on children. However, recent reports pointed out that, in children, disease characteristics, underlying causes of chronic spontaneous urticaria, and response to treatment are very similar to those reported in adults [91].

Current clinical practice and guidelines suggest that H2RAs can be considered as an adjunct to H1-antihistamines for managing chronic urticaria in children who do not achieve adequate control with optimal doses of H1-antihistamines. Optimal treatment is defined as the use of H1-antihistamines up to four times the approved dose for pediatric patients. The decision to add H2RAs to H1-antihistamine therapy should be tailored to individual clinical scenarios. Despite the lack of robust evidence, the combination of H1- and H2-antihistamines may be considered due to their relatively low cost and favorable safety profile. Identifying specific patient subgroups who may benefit from this combination therapy is crucial. However, further randomized controlled trials with larger sample sizes are needed to clarify the efficacy and safety of H2RAs as an add-on to H1-antihistamines in children with chronic urticaria.

Evidence-based recommendations

The panel provided evidence-based recommendations on the appropriate use of ARMs in pediatric practice, emphasizing the need for rational, evidence-based prescribing as summarized in Table 1. Pediatricians strongly recommended restricting PPIs to well-defined indications in children over one year of age while advocating for H2RAs as a safer alternative, particularly in neonates, infants, and critically ill children requiring short-term acid suppression. Routine PPI use in NICU and PICU settings was discouraged due to safety concerns, and SUP was advised only for high-risk patients. The panel supported the use of H2RAs for on-demand therapy, NAB, and step-down therapy from PPIs to minimize long-term acid suppression risks. Additionally, ranitidine was preferred for acid aspiration prophylaxis before surgery and as an adjunct to H1-antihistamines for chronic urticaria. It also highlights the importance of avoiding unnecessary ARM prescriptions, particularly in drug-induced dyspepsia, where on-demand H2RA use was recommended over routine co-prescription with NSAIDs or corticosteroids. These recommendations aim to optimize pediatric ARM therapy, ensuring safer and more effective clinical practices.

**Table 1:** Evidence-based recommendations on the use of gastric acid-reducing medications for children in primary care clinical settings. PPI, proton pump inhibitor; NICU, neonatal intensive care unit; H2RA, histamine-2 receptor antagonist; ARM, acid-reducing medication; SUP, stress ulcer prophylaxis; PICU, pediatric intensive care unit; GI, gastrointestinal; GERD, gastroesophageal reflux disease

Category	Guidelines
Use of PPIs in Pediatric Practices	The usage of PPIs should be judicious and limited to clear, evidence-based indications in pediatric practices.
	Routine PPI use in NICU settings should be avoided due to severe negative consequences and a lack of strong evidence.
	Avoid PPI use in children under one year unless non-pharmacological treatments fail and benefits outweigh risks.
	Prefer H2RAs (ranitidine, famotidine) over PPIs when co-administering drugs that increase kidney injury risk.
	Routine urine sample testing is recommended in children receiving PPIs for more than one week to detect early kidney damage.
Gastrointestinal Uses of H2RAs in Neonates and Infants	ARM helps prevent stress ulcers in critically ill children, particularly those on mechanical ventilation.
	Assess need for SUP in NICU/PICU patients based on coagulopathy, mechanical ventilation, or history of GI bleeding.
	Use ARM only for upper GI bleeds unrelated to Vitamin K deficiency.
	In neonates and infants, ARMs should be used only for symptomatic reflux (irritability, arching, disease progression, failure to thrive).
	Apnea alone is not a valid indication for ARM use.
	H2RAs (ranitidine, famotidine) are preferable to PPIs for SUP in critically ill NICU patients.
Gastrointestinal Uses of H2RAs in Older Children and Adolescents	Ranitidine provides rapid and effective symptom relief, making it a preferable on-demand ARM choice.
	Useful for managing nocturnal acid breakthroughs during PPI therapy.
	Effective as a step-down therapy from PPIs or as a rescue therapy when a PPI dose is missed.
Acid Aspiration Syndrome Prophylaxis	Ranitidine is preferred over PPIs for reducing acid aspiration syndrome risk in children undergoing anesthesia.
	Compared to PPIs, ranitidine is more effective in lowering stomach fluid volume and raising pH when taken before surgery.
GERD Management in Children with Asthma	Empiric ARM therapy has an inconsistent impact on asthma control; many studies show no significant improvement.
	ARM therapy should be reserved for asthmatic children with symptomatic GERD.
	No strong evidence supports routine PPI use for asymptomatic GERD in asthmatic children.
	H2RAs (ranitidine, famotidine) may be a safer alternative for GERD management in asthmatic children.
Management of Drug-Induced Dyspepsia	Drug-induced dyspepsia includes symptoms like bothersome postprandial fullness, early satiety, epigastric pain, or burning after starting a new medication.
	Routine prophylactic ARM use with dyspepsia-inducing medications is not recommended.
	ARMs should be used on an as-needed basis, initiated when symptoms arise, and discontinued within 72 hours after resolution.
	H2RAs (ranitidine, famotidine) are preferred over PPIs due to their rapid onset of action.
Non-GI Uses of H2RAs in Children	H2RAs (ranitidine, famotidine) can be used as adjuncts to H1-antihistamines for chronic urticaria in children who do not achieve adequate control with H1-antihistamines alone.
	The decision to add H2RAs to H1-antihistamine therapy should be individualized.
	H1- and H2-antihistamine combination therapy is cost-effective and has a favorable safety profile.

## Conclusions

This expert opinion paper provides a thorough analysis of ARMs' application in pediatric practice, highlighting the need for optimized and evidence-based use. By emphasizing cautious application, particularly of PPIs, and the targeted use of H2RAs in specific scenarios, this paper aims to promote the safe and effective use of ARMs in children.

The insights offered are intended to educate primary-care practitioners to adopt evidence-based practices, thereby leading to more effective and better patient care. As a resource for continuous professional development, this paper will serve as an educational tool for current and future pediatricians, helping them stay updated with best practices in ARM therapy. Future research should focus on refining prescribing practices and evaluating the role of ARMs in various pediatric conditions to build on these recommendations.
